# The predictive role of pelvic magnetic resonance in the follow up of spontaneous or induced puberty in turner syndrome

**DOI:** 10.1186/s13052-018-0458-0

**Published:** 2018-02-13

**Authors:** M. C. Maggio, A. De Pietro, P. Porcelli, F. Serraino, T. Angileri, A. Di Peri, G. Corsello

**Affiliations:** 1 Universitary Department Pro.Sa.M.I. “G. D’Alessandro”, University of Palermo, via dei Benedettini n.1, 90134 Palermo, Italy; 2 Diagnostic Operative Unit, “Villa S. Teresa Diagnostica per Immagini e Radioterapia”, Bagheria, Palermo, Italy; 3 Operative Unit of Endocrinology “Azienda Ospedali Riuniti Villa Sofia-Cervello”, ASP 6, Palermo, Italy

**Keywords:** Turner syndrome, Hypogonadism, Magnetic resonance imaging, Ultrasonography, Infertility

## Abstract

Puberty is a critical age for patients with Turner syndrome (TS): infertility is reported to be linked to karyotype and spontaneous puberty and menarche occur in approximately 30% of patients, especially in mosaicism. However, it is not always predictable considering hormonal pattern and pelvic transabdominal ultrasound scan (US).

The aim of the study is to compare the accuracy of Magnetic Resonance Imaging (MRI) and US to evaluate uterine and gonads volume, to visualize the presence of follicles and to predict spontaneous puberty and menarche in girls with TS. In a retrospective study, we evaluated 19 TS patients (age: 9–16 years), who underwent transabdominal pelvic US and pelvic MRI as required by parents. We correlated pelvic imaging with karyotype, hormonal data and pubertal outcome, and we compared US resolution to MRI.

MRI revealed a higher accuracy in the study of uterus and ovaries, and permitted to measure ovaries not visualized by US. Ovarian volume, the presence of follicles and the occurrence of spontaneous puberty were not related to the karyotype; spontaneous puberty started in one patient with a karyotype 45,X and in two patients with mosaicism (45,X/46,XX; 47,XXX/45, X). Ovarian follicles were relieved by MRI in patients with a spontaneous menarche and the persistence of menstrual cycles correlated with an ovarian volume corresponding to Tanner stage 3–4. We stress the role of MRI in the follow-up of TS adolescents, guide in the choice of the timing of treatment.

Dear Sir,

TS is the most common chromosomal aneuploidy in humans, with a prevalence of 1:2.500 females. However the real incidence in pregnancies is significantly higher, because 99% of foetuses with a 45,X karyotype undergoes to spontaneous abortion [1].

TS is characterized by a typical phenotype, associated with total or partial X chromosome monosomy, sometimes with mosaicism. Typical facies, hearth and kidney malformations, short stature and hypogonadism are characteristic. Secondary sexual characters and menses are reached in TS patients by oestroprogestinic treatment. However, an optimal timing and dose of the oestroprogestinic treatment does not guarantee uterine growth until the adult volume [2, 3]. Otherwise spontaneous puberty and menarche occur without oestroprogestinic treatment in 30% of TS patients. This favourable prognosis is described especially in mosaicism; however, it is not always predictable by karyotype, hormonal pattern and pelvic scan [4, 5].

Transabdominal pelvic US and pelvic MRI are accurate, safe and non-invasive techniques for visualizing the size and the shape of the internal genitalia in pre-pubertal and pubertal girls. US provides a quicker and accurate check up of gonads and uterus, however MRI provides measurements that are less operator-dependent [6].

Usually, ovary and uterine diameters are evaluated by transabdominal scan in TS adolescents [4]. However, US has lower sensitivity and specificity compared to laparoscopy, especially in streak gonads. Only one study [7] described the results of pelvic MRI imaging in TS. MRI showed a higher sensitivity and specificity for evaluation of ovarian follicles and uterus and a higher accuracy to predict spontaneous puberty and menarche.

The objectives of our study are to compare the accuracy of MRI vs US to evaluate uterine and gonads volume; to visualize follicles and to predict spontaneous puberty and menarche in our patients; to highlight the role of MRI in the follow-up of TS adolescents and in the choice of treatment line and timing.

We present a retrospective study on the evaluation and follow up, over a 6 years period, of 19 patients with TS, diagnosed by karyotype. Patients age was: 9–16 years; bone age (by Greulich and Pyle method) was: 12.4 ± 2.2 years; all the patients underwent transabdominal pelvic US and pelvic MRI, as required by parents. We compared ovarian and uterine size, follicles number and volume evaluated by transabdominal US vs by MRI and we correlated pelvic imaging with karyotype and hormonal data (FSH, LH; Prolactin, TSH, fT3, fT4, IGF-1).

In all the patients a standard karyotype was obtained by lymphocytes culture of peripheral blood and revealed the following karyotypes: 45,X in 7 patients; 45,X/46,XX in 1; 45,X/47,XXX in 1; 45,X/46,XY in 1; deletion of the short arm of X chromosome in 1; 45,X/46 del(Xp) mosaicism in 1; mosaicism 45,X/46,X,i(Xq) in 4; mosaicism 45,X/46,X,r(X) in 3.

All the patients received or are currently treated with GH (0.03–0.04 mg/kg/day) with a significant improvement of growth. Three patients developed hypothyroidism secondary to autoimmune thyroiditis and received L-thyroxine with an adequate normalization of the hormonal pattern. None developed Celiac disease during the follow up. Two patients had kidney malformations, with no impairment of the renal function. None had cardiac malformations with hemodynamic significance. Six patients were treated with transdermal oestrogens or with 17β-oestradiol plus progesterone, with a regular menstrual bleeding.

Three patients had a spontaneous puberty and menarche, reached without oestrogens and still have regular menses. Transabdominal pelvic US was realized by a 3.5–5 MHz probe. We evaluated uterine and ovarian size, as well as the presence and number of ovarian follicles.

MRI was conducted without contrast, with spin-echo (SE), FAST-SE, T1 and T2-weighted images in axial, coronal and sagittal sections. All the patients were studied by the same experienced specialist in radiology.

The pelvis was scanned in a 1.5 TL in axial, coronal and sagittal planes (Philips 1.5 TL), high performance 4-channels body phased array coils. T1w, T2/TSE/SPIR, DW1 sequences were performed. T1w and T2w sequences, the best choice to study ovaries, were performed with slice thickness of 5 mm. T2w sequence, considered the gold standard to study uterus and gonads, was performed also with slice thickness of 3–4 mm in patients with a hypoplastic uterus. MRI defined the following uterine measurements (see Table 4): TD; APD; fundus/cervix ratio; endometrial thickness. Furthermore, MRI defined: right ovarian volume; left ovarian volume; right ovarian TD; left ovarian TD; right ovarian APD; left ovarian APD.

Uterine and ovarian volumes were calculated using the following formula: Volume (ml) = LD (cm) x TD (cm) x APD (cm) × 0,5233 [7]. The fundus/cervix ratio was calculated by dividing the longitudinal diameters of the corpus with the longitudinal diameter of the cervix.

As reference of the physiological uterine and ovarian size we considered the population of healthy girls described in the work of K. Holm et al. [8] (see Table 1).

**Table 1:** Uterus and ovaries US measurements in relation to breast development, in healthy girls (from “Holm K, Laursen EM, Brocks V, Müller J: Pubertal maturation of the internal genitalia: an ultrasound evaluation of 166 healthy girls. Ultrasound in Obstetrics and Gynecology 1995;6,175-181”, modified)

Uterine US measurements: Median (range)	Uterine volume (ml)	Ovarian volume (ml)
B1	1.6 (07–7.9)	1.2 (0.5–5.1)
B2	2.8 (1.3–8.1)	2.2 (1–4.6)
B3	8 (2–18)	4.1 (1.9–8.6)
B4	37 (11–56)	6.2 (1.3–28)
B5	43 (12–82)	7.3 (1.9–23)
≥19 years	61 (37–130)	7.6 (2.9–37)

We have obtained consent to publish from the participant (or legal parent or guardian for children) to report individual patient data.

Hormonal parameters are summarized in Table 2.

**Table 2:** Hormonal parameters

FSH	25.74 ± 38.76 mIU/ml
LH	4.07 ± 4.46 mIU/ml
Prolactin	9.86 ± 5.76 ng/ml
IGF-1	364.9 ± 206.5 ng/ml

US and MRI measurements are summarized in Tables 3 and 4, respectively.

**Table 3:** uterus and ovaries US measurements in TS

Uterine US measurements	M	SDS	Range
LD	4.33	1.92	2.00–8.13
Corpus	2.55	1.45	1.00–5.42
Collus	1.70	0.56	1.00–2.71
Fundus/Cervix	1.39	0.50	1.00–2.00
Right ovarian LD (ml)	2.6	0.1	2.4–2.70
Left ovarian LD (ml)	2.77	0.45	1.87–3.20

**Table 4:** uterus and ovaries MRI measurements in TS

Uterine MRI measurements	M	SDS	Range
TD	2.32	1.32	0.60–4.30
APD	2.05	1.17	0.50–3.60
Corpus	2.83	1.52	0,90–5.00
Collus	1.79	0.69	0.60–3.00
Fundus/cervix	1.55	0,54	1.00–2.50
endometrial thickness	6.90	3.90	3.00–16.00
Right ovarian volume (ml)	3.16	3.44	1.3–8.3
Left ovarian volume (ml)	5.40	4.10	2.5–8.3
Right ovarian TD (mm)	1.48	0.43	1.00–2.00
Left ovarian TD (mm)	1.3	0.14	1.20–1.40
Right ovarian APD (mm)	2.23	0.76	1.30–3.10
Left ovarian APD (mm)	2.55	1.34	1.60–3.50

The fundus/cervix ratio detected by MRI was higher (1.55 ± 0.54) than the ratio measured by US (1.39 ± 0.50). The endometrial thickness was detected in 11/19 patients (58%), while endometrium was not visible in the remaining 8. The ovarian APD detected by MRI was not significantly different than the ovarian LD measured by US; however, in two patients, ovaries were visualized by MRI and not by US. The ovarian volume and the follicles number were not related to karyotype (mosaicism or 45,X); 3 patients showed a spontaneous start and progression of puberty: one with 45,X; two with mosaicism (45,X/46, XX; 47,XXX/45, X). The presence of ovarian follicles was relieved by MRI in patients with a spontaneous menarche and the persistence of menstrual cycles, and it was correlated with an ovarian volume > 7 cm (see Fig. 1).

**Fig. 1:**
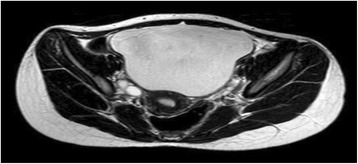
MRI imaging of a patient with spontaneous menarche

Gonadotropins levels, especially FSH, were negatively correlated with US and MRI measurements of uterine and ovarian size, without reaching the statistical significance. Besides FSH and LH showed an inverse correlation with fundus/cervix ratio measured by US and by MRI; FSH and LH maintained an inverse correlation with endometrial thickness, confirmed by lower levels of gonadotropins in patients with spontaneous menarche.

MRI revealed a higher definition of imaging for uterus and ovaries, defining volume and morphology, follicular volume, endometrial thickness, uterine body/neck ratio (see Fig. 2).

**Fig. 2:**
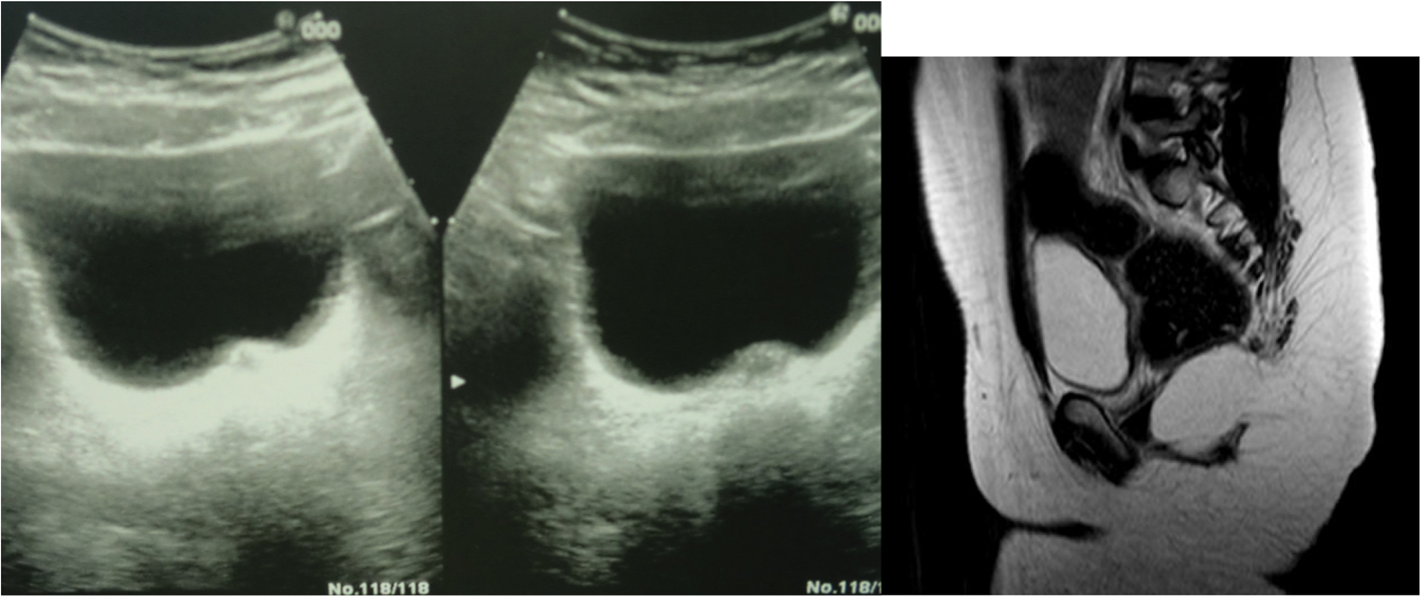
US and MRI immaging in a patient with hypoplastic uterus

The endometrial thickness was measured with a higher precision by MRI than by US.

Puberty is a critical age for patients with TS, with problems related to possible spontaneous puberty and menarche, to oestrogen induction and progression of puberty, and to future fertility.

We did not find a direct correlation between karyotype and spontaneous menarche and/or puberty. The karyotype of the patients with spontaneous puberty and/or menarche varied from 45,X to mosaicism.

Transabdominal pelvic US is an accurate, low cost and widely available technique [8]. In a routine pelvic imaging follow up of TS patients, US is the universally available and suitable recommended technique. Nevertheless, US has a low reproducibility especially if repeated by different radiologists. However, as previously evidenced [7], MRI has a higher definition of uterus and ovaries volume and morphology; in fact, it shows a higher sensitivity in the measurement of uterus and ovaries volumes, and in the recognition of follicles and in the evidence and measurement of endometrial thickness. In our study, in fact, MRI showed ovaries in two patients, otherwise not detected by US. The ovaries imaging well correlates with gonadotropins levels, both in patients with spontaneous or induced pubertal development. Furthermore, ovaries volume and spontaneous puberty does not correlate with karyotype in our patients.

MRI can help in the study of ovaries and shows a higher sensitivity in their evaluation, when they are behind the uterus, when bladder is empty, when meteorism is present. MRI is the best choice to follow TS patients with mosaicism including Y or fragments of Y chromosome. In fact, this karyotype is a risk factor of dysgerminoma in dysgenic gonads [9].

MRI in T2w (in sagittal, coronal and axial planes) has a higher definition than US when the uterus is hypoplastic. MRI could represent a future gold standard in the follow up of adolescent patients with TS: in fact, it is able to view small ovaries and follicles otherwise not found by transabdominal pelvic US.

However, we must consider the significantly higher costs of MRI compared to US and the difficulties to repeat the exam frequently in patients who need a follow-up, as TS girls. In younger patients the difficulties to perform MRI are often linked to the need to do a sedation of the patient; for this reason, we organized MRI only for patients who did not require a sedation.

Therefore, we suggest performing MRI almost in pubertal age and comparing MRI with US measurements and hormonal data. The evidence of well defined ovaries, associated with normal levels of FSH, LH, oestradiol could be a favourable prognostic element of spontaneous puberty. The follow-up can be assured by US, monitoring the efficacy of treatment and the timing of addiction of progesterone to oestrogens.

However, MRI can integrate the endocrine follow-up, help to distinguish patients with potential spontaneous pubertal maturation and to choose the appropriate time to begin estrogens treatment, avoiding unnecessary drugs. MRI is a useful guide in the choice of drugs and of their correct management timing in the follow-up of TS adolescents. These findings can improve the follow-up of these patients and select those with a prognosis of possible fertility [10].

In addition, MRI could be a milestone in the diagnosis and identification of TS patients who are candidate to ovary cortex biopsy.

These critical points require a multidisciplinary approach, with still open questions on the endocrine and psychological follow-up of TS patients.

## Abbreviations

APD: Anteroposterior diameter
LD: Longitudinal diameter
MRI: Magnetic resonance imaging
SE: Spin-echo
TD: Transverse diameter
TS: Turner syndrome
US: Ultrasound scan
